# Bullous drug eruption with low dose methotrexate

**DOI:** 10.14744/nci.2020.83798

**Published:** 2021-12-31

**Authors:** Tuba Yuce Inel

**Affiliations:** Division of Rheumatology, Department of Internal Diseases, Dokuz Eylul University Faculty of Medicine, Izmir, Turkey

**Figure 1.**
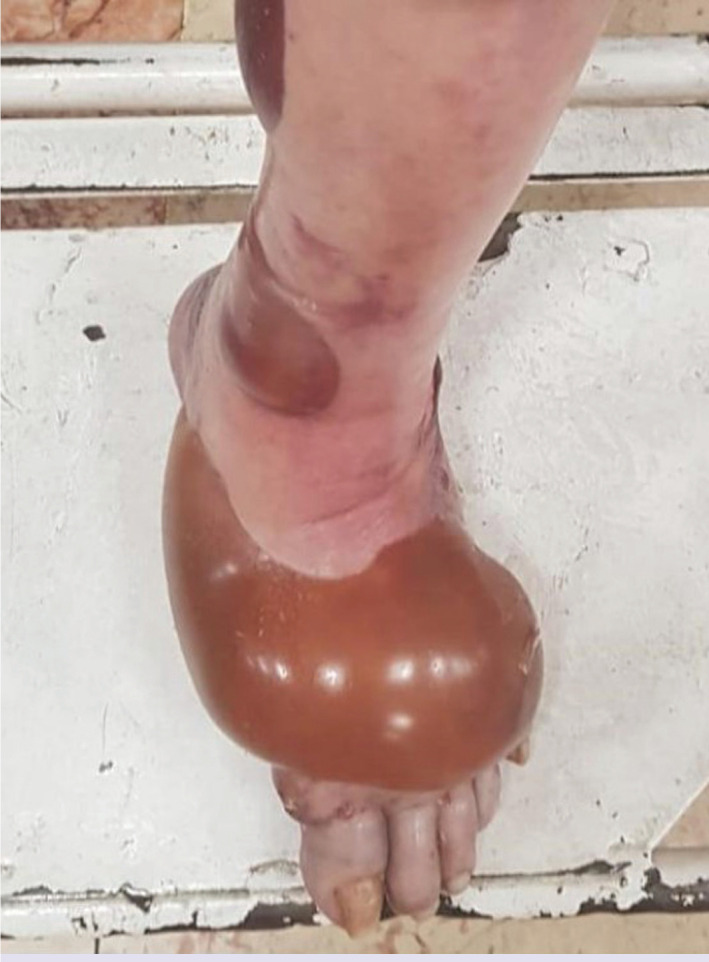
Bullous lesions on the left leg and dorsal aspect of the foot.

**A** 71-year-old woman was referred by an ophthalmologist who treated her corneal melt. Although she had erosive rheumatoid arthritis, she was not followed up for 5 years. After the diagnosis was confirmed by clinical and radiological, methotrexate (MTX) 7.5 mg/week was started because of the arthritis in her right wrist and metacarpophalangeal joints. Two days later, bullous lesions developed on the dorsal aspect of the left foot, and behind the left leg (Fig. 1). She did not have a fever or mucosal lesions. No drug-related cytopenia was observed. Levels of albumin, creatinine, and liver function tests were normal, proteinuria was not detected. Anti-nuclear antibody, anti-neutrophil cytoplasmic antibody, complements (C3–C4), and hepatitis serology were negative. MTX treatment was discontinued. Skin biopsy was performed for the differential diagnosis. Direct immunofluorescence examination of skin biopsy was negative and reported as bullous drug eruption. After the intact bullae were opened with a sterile injector, they were dressed with fusidic acid and ethacridine lactate. Meropenem and linezolid antibiotherapy were given to the patient who developed wound infection. Two weeks later, healthy re-epithelization was observed at the wound site.

Cutaneous side effects of MTX are secondary to hypersensitivity reactions or induction of apoptotic cell death. Bullous acral erythema may develop with high dose MTX treatment which occurs 1–21 days after chemotherapy administration [1]. This reaction is usually seen at high doses, but also there are few reports indicating that it is also seen at low doses [2]. It should be kept in mind that bullous drug eruption may be seen with low dose MTX, possibly as a result of an idiosyncratic reaction.
